# Senescence marker protein 30 in acute liver failure: validation of a mass spectrometry proteomics assay

**DOI:** 10.1186/1471-230X-8-17

**Published:** 2008-05-28

**Authors:** Sa Lv, Jiang-hua Wang, Feng Liu, Yan Gao, Ran Fei, Shao-cai Du, Lai Wei

**Affiliations:** 1 Peking University People's Hospital, Peking University Hepatology Institute, Beijing 100044, PR China

## Abstract

**Background:**

Our previous proteomic study showed that the senescence marker protein (SMP30) is selectively present in the plasma of a murine model of acute liver failure (ALF). The aim of this study was to validate this SMP30 expression in the plasma and liver tissues of mice and humans with ALF.

**Methods:**

After the proteomic analysis of plasma from a murine model of D-galactosamine/lipopolysaccharide (GalN/LPS)-induced ALF by two-dimensional electrophoresis (2-DE) and mass spectrometry, the expression levels of SMP30 in the plasma and liver tissues were validated by western blot and RT-PCR analyses. These results were then confirmed in plasma samples from humans.

**Results:**

These data validate the results of 2-DE, and western blot showed that SMP30 protein levels were only elevated in the plasma of ALF mice. Further analysis revealed that GalN/LPS induced the downregulation of SMP30 protein levels in liver tissues (by approximately 25% and 16% in the GalN/LPS-treated mice and in the treated mice that survived, respectively; *P *< 0.01). Hepatic SMP30 mRNA levels decreased by about 90% only in the mice that survived the GalN/LPS treatment. Importantly, plasma obtained from patients with ALF also contained higher levels of SMP30, about (3.65 ± 0.34) times those observed in healthy volunteers.

**Conclusion:**

This study shows that SMP30 is not only a potential biomarker for the diagnosis and even prognosis of ALF. It also plays a very important role in a self-protective mechanism in survival and participates in the pathophysiological processes of ALF.

## Background

Comparative proteomics has provided new insights into the mechanisms and processes of liver disease. Many candidate biomarkers have been discovered that are associated with hepatocarcinoma, viral hepatitis, or liver cirrhosis [1-4]. However, the proteins identified in these studies should be examined by other techniques to confirm their expression patterns. Because many studies involve animal experiments, their results should also be validated in human samples.

In a previous study, we established a murine model of acute liver failure (ALF) by the administration of D-galactosamine/lipopolysaccharide (GalN/LPS). Using proteomic analysis with two-dimensional electrophoresis (2-DE) and mass spectrometry, we identified 10 protein molecules with altered expression in the plasma of ALF mice [5]. Among these candidate proteins, one protein molecule, senescence marker protein 30 (SMP30), caught our interest because it only appeared on the 2-D gels of ALF mouse plasma proteins.

SMP30, an important marker of aging, is a novel protein expressed mainly in hepatocytes and renal tubule epithelia [6]. Because its structure is highly conserved in various animal species [7] and its expression is tissue-specific in hepatocytes, SMP30 has been suggested to play a pivotal role in the liver. Many researchers have identified the multifunctional roles of SMP30, such as in the maintenance of intracellular Ca^2+ ^homeostasis [8] and the regulation of intracellular signal processes [9]. SMP30 suppressed cell proliferation in regenerating rat liver and in cloned hepatoma cells by inhibiting DNA and RNA syntheses in the nucleus [10-12]. SMP30 also suppressed cell death and apoptosis in cloned rat hepatoma H4-II-E cells [13].

As a liver-specific protein, elevated plasma levels of SMP30 have not been reported in ALF. The aim of this study was twofold: first, to validate the high levels of SMP30 protein in the plasma of ALF mice and humans; and second, to examine the expression of SMP30 protein and mRNA levels in the liver tissues of ALF mice and to evaluate the potential role of SMP30 in liver failure.

## Methods

### Animals and treatment

Male BALB/c mice, 18–22 g, were obtained from the Academy of Military Medical Sciences (Beijing, China). They were housed and cared for in rooms maintained at a constant temperature and humidity. Food and water were allowed *ad libitum*. Food was withdrawn the evening before the experiment. All animal experimental procedures were approved by the Ethics Committee of Peking University People's Hospital before the commencement of the study.

All mice were randomly divided into eight groups (n = 8 per group), and were given intraperitoneal injections of D-galactosamine (600 mg/kg body weight; Sigma, Saint Louis, USA) and lipopolysaccharide (LPS; 8 μg/kg body weight; Sigma), as described previously [5]. Control animals were intraperitoneally injected with GalN, LPS, or saline. Plasma and liver tissue samples were obtained 5 and 24 h after injection and were stored at -80°C until analysis. Plasma levels of alanine aminotransferase (ALT) and T-bilirubin (TBiL), measured with a 7170A automatic analyzer (HITACHI, Japan), were used to assess the extent of liver injury.

In summary, the eight groups were (1): 5 h after saline injection; (2): 5 h after LPS injection; (3): 5 h after GalN injection; (4): 5 h after GalN/LPS injection; (5): 24 h after saline injection; (6): 24 h after LPS injection; (7): 24 h after GalN injection; (8): 24 h after GalN/LPS injection.

### Plasma protein proteomics

The plasma proteomics protocol was as described in our previous study [5]. In brief, plasma samples were pooled and precipitated with cold acetone, then dissolved in buffer containing 7 M urea, 2 M thiourea, 4% CHAPS, 1 mM EDTA, 50 mM DTT, and 1 mM PMSF. Total protein concentrations were determined with the Bradford method. The protein (2 mg) was solubilized in rehydration buffer containing 6 M urea, 2% CHAPS, 20 mM DTT, 0.5% IPG buffer, and a trace amount of bromophenol blue. Isoelectric focusing (IEF) was performed with commercially available preformed immobilized pH gradients (nonlinear, pH 3–7, 24 cm; Amersham, Sweden) using an IPGphor Isoelectric Focusing System (Amersham). Following a three-step equilibration, the IPG strips were positioned on 12% polyacrylamide gels and stained with Coomassie Brilliant Blue G-250 [5]. After they had been scanned with Labscan (Amersham), the digitized images of the stained gels were analyzed using ImageMaster 2-D ver. 5.1 software (Amersham). Only spots that were present on the experimental gels and were altered at least twofold compared with the control gels were considered to be significant and were identified by mass spectrometry. After digestion with trypsin (sequencing grade; Roche, USA), the peptides were processed by liquid chromatography-electrospray ionization-tandem mass spectrometry (LC-ESI-MS/MS). MS/MS spectra were compared with the NCBI nr-databank using the SEQUEST algorithm.

### Western blot (WB) analysis of SMP30

Liver tissues were homogenized in lysis buffer (50 mM Tris-Cl [pH 8.0], 1% NP-40, 150 mM NaCl, 0.1% SDS, 0.02% sodium azide, and 100 μg/mL PMSF). Plasma proteins (10 μg) or tissue proteins (50 μg) were separated electrophoretically on SDS-PAGE (12%) and electrotransferred onto a nitrocellulose membrane (40 V overnight). The membranes were blocked with nonfat dried milk in TBS containing 0.2% Tween-20 (TTBS) for 1 h at room temperature. Membranes were then incubated overnight at 4°C with goat polyclonal anti-SMP30 antibody (diluted 1: 300 for plasma and 1:500 for tissues; Santa Cruz Biotechnology, USA) or rabbit polyclonal anti-β-actin antibody (diluted 1:1000; Santa Cruz Biotechnology). After they had been washed three times in TTBS, the membranes were reacted with horseradish-peroxidase-labeled secondary antibody (diluted 1:3000; Santa Cruz Biotechnology) for 1 h at room temperature. Immunodetection was performed with the ECL-Plus kit (Pierce Biotechnology, USA), according to the manufacturer's instructions.

### Reverse transcriptase-PCR for SMP30 mRNA

Total RNA was isolated from liver tissues with TRIzol Reagent (Invitrogen, USA). First-strand cDNA was generated from 500 ng of total RNA using an oligo (dT) primer and SuperScript III reverse transcriptase (Invitrogen). PCR was performed using *Taq *DNA polymerase (Qiagen, Valencia, USA) and oligonucleotide primers for mouse SMP30 (forward 5'-GGAGGCTATGTTGCCACCATTGGAAC-3', reverse 5'-TTCCCTCCAAAGCAGCATGAAGTTGTTTTA-3', amplicon size 560 bp) and glyceraldehyde 3-phosphate dehydrogenase (GAPDH; forward 5'-GTGAAGGTCGGTGTGAACGGAT-3', reverse 5'-GCATCCTGCTTCACCACCTTCTT-3', amplicon size 788 bp). The samples were incubated at 94°C for 2 min, and then amplified for 20 cycles of denaturation for 30 s at 94°C, annealing for 30 s at 60°C, and extension for 59 s at 72°C. The PCR products were analyzed by fractionation on a 1.2% agarose gel and visualized with ethidium bromide staining. Images were captured using a gel documentation system (Quantity One, Bio-Rad, USA).

### Patient protocol

Plasma samples were obtained from four patients diagnosed with ALF. ALF was defined as liver failure with jaundice and an international normalized ratio value of > 1.5 in patients without pre-existing chronic liver disease and with an illness of less than 26 weeks duration. ALF resulted from hepatitis B virus infection in two patients and from antituberculosis drugs in the remaining two patients. Control plasma samples were obtained from four healthy volunteers. There were no significant differences in age or sex between the ALF patient group and the control group. All samples were stored at -80°C until further WB analysis. This study was conducted in agreement with the Ethics Committee of Peking University People's Hospital.

### Statistical analysis

SPSS ver. 10.0 was used for the statistical analyses. All data were analyzed using analysis of variance (ANOVA) followed by a least-squares difference test. The relationships between SMP30 levels in liver tissues and the ALT, TBiL in plasma of animal model were analyzed by the Pearson correlation test. *P *values of < 0.05 were considered significant. All data are presented as the mean ± S.E.

## Results

### SMP30 only elevated in plasma of ALF mice according to 2-DE

**Figure 1:**
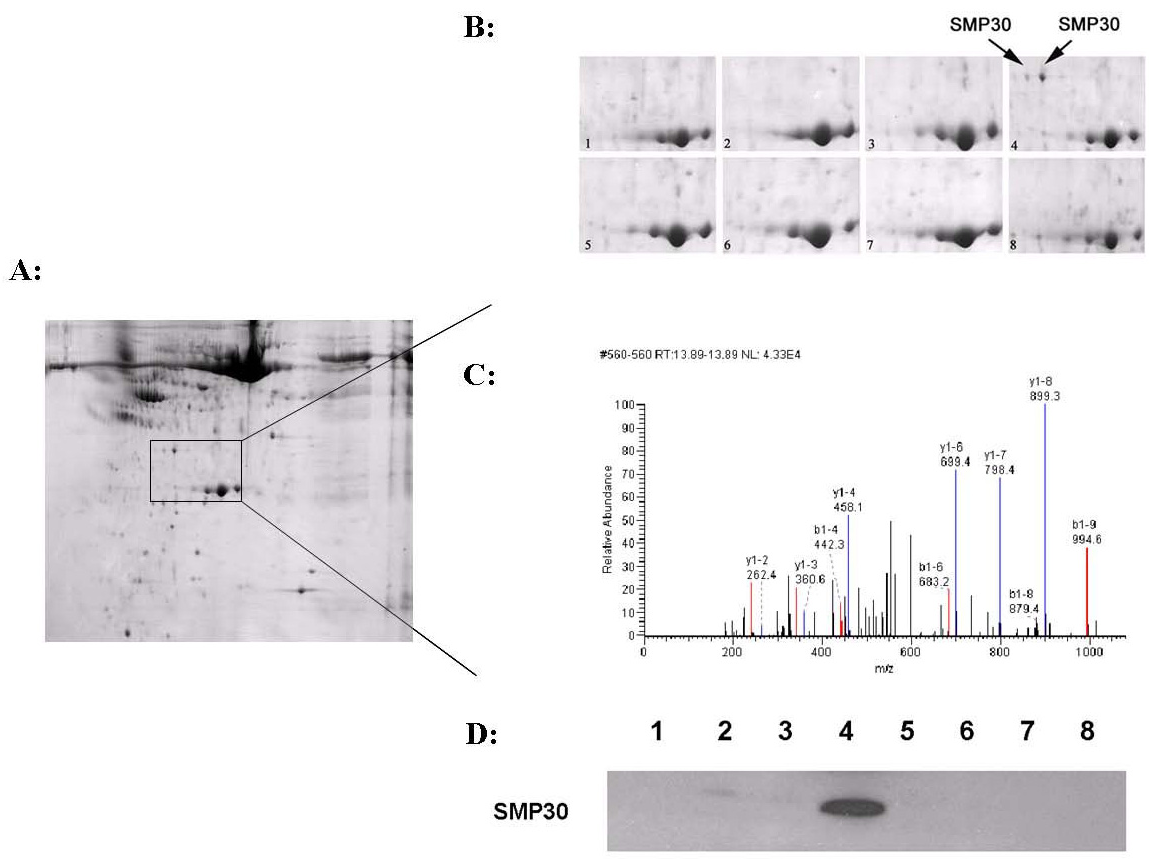
**Plasma SMP30 levels were only elevated in mice with ALF**. A: 2-D gel of murine plasma proteins. B: Magnified regions of 2-D gel of murine plasma proteins. The results shown are representative of three independent experiments. The protein spot corresponding to SMP30 is indicated with an arrow. C: Spectrum from LC-ESI-MS/MS analysis of the trypsin digestion products of senescence marker protein 30 (SMP30). D: WB analysis of plasma SMP30 levels in GalN/LPS-treated mice. Lane and gel 1: 5 h after saline injection; lane and gel 2: 5 h after LPS injection; lane and gel 3: 5 h after GalN injection; lane and gel 4: 5 h after GalN/LPS injection; lane and gel 5: 24 h after saline injection; lane and gel 6: 24 h after LPS injection; lane and gel 7: 24 h after GalN injection; lane and gel 8: 24 h after GalN/LPS injection.

Coinjection of GalN/LPS successfully induced the ALF animal model (group 4) and about 10% of these mice survived (group 8). Plasma ALT and TBiL levels increased significantly at 5 hours and 24 hours after GalN/LPS injection (Table 1). We analyzed the plasma proteins of the ALF mice and the other controls by 2-DE. Differentially expressed protein spots were identified with software analysis. Of these proteins, the expression of only two protein spots was elevated in the plasma of ALF mice. When identified by MS, these two proteins spots were the same protein molecule, SMP30 (Figure 1A, B, C), with a molecular weight of 33.4 kDa and an isoelectric point (pI) of 5.16.

**Table 1:** Serum ALT and TBiL levels in mice

**group**	**saline**	**LPS**	**GalN**	**GalN/LPS**	**saline**	**LPS**	**GalN**	**GalN/LPS**
		
	**5 h**				**24 h**			
**ALT(U/L)**	45.25 ± 9.64	41.50 ± 10.11	62.13 ± 23.93	2034.00 ± 343.68*	46.63 ± 9.75	42.13 ± 9.92	57.63 ± 12.38	1655.50 ± 722.62*
**TBiL(μmol/L)**	0.34 ± 0.29	0.49 ± 0.21	0.36 ± 0.29	4.71 ± 0.65*	0.53 ± 0.33	0.50 ± 0.15	0.48 ± 0.27	3.53 ± 1.72*

*: *P *< 0.01 vs control group

### Validation of GalN/LPS-induced SMP30 levels in plasma by WB

SMP30 was selectively expressed in response to GalN/LPS treatment, as determined by 2-DE. This result was confirmed by WB, as shown in Figure 1D. Plasma SMP30 levels were only elevated in ALF mice treated with GalN/LPS (group 4) compared with those of the control groups. Moreover, plasma SMP30 levels returned to the control level in surviving animals (group 8). This result parallels that obtained with 2-DE.

### GalN/LPS-induced expression of SMP30 protein and mRNA in liver tissues

**Figure 2:**
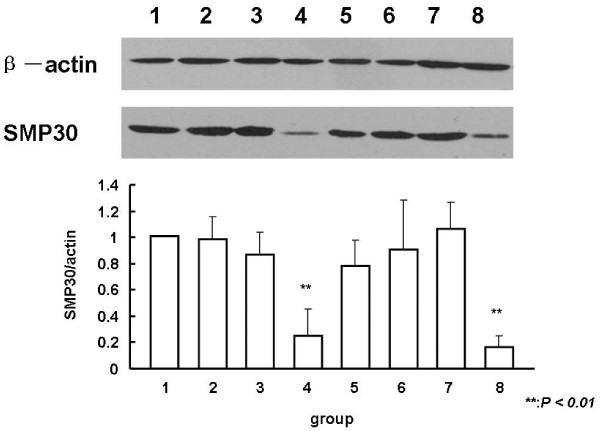
**WB analysis of liver tissue SMP30 levels in GalN/LPS-treated mice**. Lane 1: 5 h after saline injection; lane 2: 5 h after LPS injection; lane 3: 5 h after GalN injection; lane 4: 5 h after GalN/LPS injection; lane 5: 24 h after saline injection; lane 6: 24 h after LPS injection; lane 7: 24 h after GalN injection; lane 8: 24 h after GalN/LPS injection. SMP30 levels were significantly reduced in the liver tissues of mice treated with GalN/LPS compared with those of the control groups (*P *< 0.01). There was no significant difference between the SMP30 protein levels of groups 4 and 8 (*P *> 0.05).

To investigate whether there is a relationship between plasma SMP30 protein levels and the expression of this protein in liver tissues, WB analysis of liver-specific SMP30 expression was performed. As shown in Figure 2, SMP30 protein levels were significantly reduced in the liver tissues of GalN/LPS-treated mice compared with the tissues of their control counterparts (approximately 25% and 16% on 4 and 8 group respectively, *P*_*4,8 *_< 0.01). There was no significant difference in the liver SMP30 protein levels of groups 4 and 8 (*P *> 0.05).

In order to investigate the relationship between expression of SMP30 in liver tissues and ALT, TBiL levels in plasma of animal model, we calculated Pearson correlation coefficients. Correlation analysis revealed significant negative correlations between SMP30 levels and ALT, TBiL (*P *< 0.01; r = -0.413 and -0.574, respectively).

**Figure 3:**
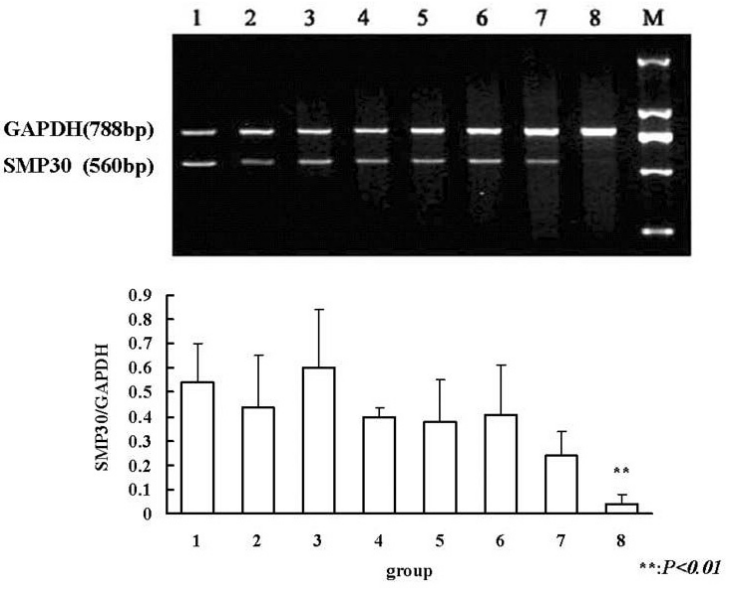
**RT-PCR analysis of liver tissue SMP30 mRNA levels in GalN/LPS-treated mice**. Lane 1: 5 h after saline injection; lane 2: 5 h after LPS injection; lane 3: 5 h after GalN injection; lane 4: 5 h after GalN/LPS injection; lane 5: 24 h after saline injection; lane 6: 24 h after LPS injection; lane 7: 24 h after GalN injection group; lane 8: 24 h after GalN/LPS injection. SMP30 mRNA levels were significantly reduced in liver tissues only at 24 h after GalN/LPS administration (*P*_*8 *_< 0.01 *vs *other control groups).

RT-PCR revealed that SMP30 mRNA levels were significantly reduced about 90% in liver tissues 24 h after GalN/LPS treatment, but were unchanged 5 h after its administration (Figure 3, *P*_*8 *_< 0.01).

### Elevated plasma SMP30 levels in ALF patients

**Figure 4:**
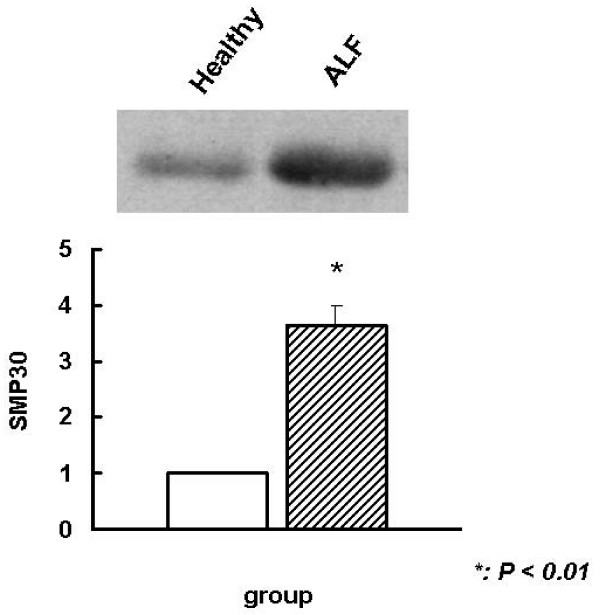
**WB analysis of plasma SMP30 levels in ALF patients**. Plasma from healthy volunteers was used as the control. n = 4 for both groups. SMP30 protein levels in ALF patients were significantly elevated, about 3.65 ± 0.34 times, compared with those in healthy volunteers (*P *< 0.01).

The expression of SMP30 in ALF mice caught our interest. Therefore, we determined whether SMP30 levels are altered in the plasma of patients with ALF. Plasma proteins from patients with ALF and healthy volunteers were analyzed by WB. As can be seen in Figure 4, SMP30 protein levels in ALF patients were significantly elevated, about 3.65 ± 0.34 times, compared with those of the healthy volunteers (*P *< 0.01).

## Discussion

Our data validate previous findings and show that (1) plasma SMP30 protein levels are significantly elevated in mice and patients with ALF; (2) liver SMP30 protein levels are significantly reduced in GalN/LPS-treated mice; and (3) liver SMP30 mRNA levels are only reduced in those animals that survive treatment with GalN/LPS.

In this study, we have demonstrated for the first time, using a comparative proteomics approach, that SMP30 levels were only significantly elevated in the plasma of our ALF animal model. Furthermore, we verified by WB that the levels of SMP30 were increased in the plasma of ALF mice but not in the plasma of surviving animals. This result is consistent with the results of 2-DE. The results of the animal experiments prompted us to determine whether there are similar changes in humans. As we expected, plasma SMP30 levels were significantly elevated in ALF patients compared with those in healthy volunteers, according to WB analysis. These consistent results in animals and humans suggest that plasma SMP30 is a potential biomarker of liver injury and may even be useful in ALF diagnosis. However, the number of patients was small, so this result should be considered preliminary and additional research with clinical samples should be undertaken.

As a tissue-specific protein, SMP30 is synthesized and expressed predominantly in the liver tissues of humans and mice [6,7,14]. Was its abnormal increase in plasma secondarily induced by the changes in its expression in the liver? Conversely, WB analysis demonstrated that SMP30 protein levels were reduced in the livers of mice treated with GalN/LPS. We speculated that the reasons for this discrepancy in the plasma and liver levels of SMP30 in ALF mice included (1) an increase in the membrane permeability of damaged hepatocytes and the necrosis of hepatocytes, allowing the release of SMP30 into the blood [15]; (2) a decrease in the clearance and degradation of SMP30 from the plasma, resulting from severe hepatic injury; and (3) some factors that stimulated other tissues to release SMP30 into the plasma during the progress of ALF. Importantly, we found that plasma SMP30 levels returned to the control level in the surviving animals (group 8), although their liver SMP30 protein levels had decreased. This phenomenon indicates that plasma SMP30 is probably not only a potential biomarker for the diagnosis of ALF, but may also be useful in assessing prognoses in severe hepatic injury.

In this study, we found that SMP30 protein levels were reduced in the liver tissues of GalN/LPS-treated mice. In order to investigate the relationship between expression of SMP30 in liver tissues and the extent of liver injury, we calculated Pearson correlation coefficients. Correlation analysis revealed significant negative correlations between SMP30 levels in liver tissues and ALT, TBiL levels in plasma (*P *< 0.01). These negative correlations indicate that when severe liver injury develops, the downregulation of SMP30 in liver tissues would allow tissue regeneration. Other researchers have also found that liver SMP30 protein levels decreased when animals were administered carbon tetrachloride [16,17], phenobarbital [18], or LPS [19]. Liver tissue is the most important target organ of chemical injury. Hepatocytes can be induced to proliferate to ensure normal liver function. Many previous studies have found that the overexpression of SMP30 suppresses the proliferation of liver cells [17,20]. In the process of hepatocyte necrosis, this decrease in SMP30 is perhaps a self-protective mechanism that reduces its effect of inhibiting proliferation. SMP30 mRNA levels were especially significantly reduced in the mice that survived treatment with GalN/LPS. This result suggests that the decreased expression of liver SMP30 is controlled at the level of transcription. More importantly, the liver tissues of the mice that survived treatment with GalN/LPS displayed hepatocyte proliferation. Therefore, there may be factors that regulate the expression of SMP30 mRNA, and the consequent low levels of SMP30 protein participate in the regulation of hepatocyte proliferation. Thus, a minority of mice recover spontaneously.

## Conclusion

This study achieved its primary purpose of validating the expression of one candidate biomarker discovered using 2-DE and LC-ESI-MS/MS. Comparative proteomic analysis and continuing validation research led to the identification of a novel protein, SMP30, which is not only a potential biomarker of ALF diagnosis even prognosis, but may also play a very important role in the self-protective mechanism that facilitates survival in ALF.

## Competing interests

The authors declare that they have no competing interests.

## Authors' contributions

SL, J–HW and LW participated in the conception and design of the study, drafted the manuscript. SL, J–HW, FL and RF carried out practical performance. SL and YG analyzed the data. S–CD collected the plasma samples of patients. All authors have read and approved the final manuscript.

## Pre-publication history

The pre-publication history for this paper can be accessed here:



## References

[B1] Feng JT, Shang S, Beretta L (2006). Proteomics for the early detection and treatment of hepatocellular carcinoma. Oncogene.

[B2] Low TY, Leow CK, Salto-Tellez M, Chung MC (2004). A proteomic analysis of thioacetamide-induced hepatotoxicity and cirrhosis in rat livers. Proteomics.

[B3] He QY, Lau GK, Zhou Y, Yuen ST, Lin MC, Kung HF, Chiu JF (2003). Serum biomarkers of hepatitis B virus infected liver inflammation: a proteomic study. Proteomics.

[B4] Parent R, Beretta L (2005). Proteomics in the study of liver pathology. Hepatology.

[B5] Lv S, Wei L, Wang JH, Wang JY, Liu F (2007). Identification of novel molecular candidates for acute liver failure in plasma of BALB/c murine model. J Proteome Res.

[B6] Fujita T, Uchida K, Maruyama N (1992). Purification of senescence marker protein-30 (SMP30) and its androgen-independent decrease with age in the rat liver. Biochim Biophys Acta.

[B7] Fujita T, Mandel JL, Shirasawa T, Hino O, Shirai T, Maruyama N (1995). Isolation of cDNA clone encoding human homologue of senescence marker protein-30 (SMP30) and its location on the X chromosome. Biochim Biophys Acta.

[B8] Fujita T, Inoue H, Kitamura T, Sato N, Shimosawa T, Maruyama N (1998). Senescence marker protein-30 (SMP30) rescues cell death by enhancing plasma membrane Ca^2+^-pumping activity in Hep G2 cells. Biochem Biophys Res Commun.

[B9] Matsuyama S, Kitamura T, Enomoto N, Fujita T, Ishigami A, Handa S, Maruyama N, Zheng D, Ikejima K, Takei Y, Sato N (2004). Senescence marker protein-30 regulates Akt activity and contributes to cell survival in Hep G2 cells. Biochem Biophys Res Commun.

[B10] Tsurusaki Y, Yamaguchi M (2002). Suppressive role of endogenous regucalcin in the enhancement of deoxyribonucleic acid synthesis activity in the nucleus of regenerating rat liver. J Cell Biochem.

[B11] Tsurusaki Y, Yamaguchi M (2002). Role of endogenous regucalcin in nuclear regulation of regenerating rat liver: suppression of the enhanced ribonucleic acid synthesis activity. J Cell Biochem.

[B12] Misawa H, Inagaki S, Yamaguchi M (2001). Suppression of cell proliferation and deoxyribonucleic acid synthesis in the cloned rat hepatoma H4-II-E cells overexpressing regucalcin. J Cell Biochem.

[B13] Izumi T, Yamaguchi M (2004). Overexpression of regucalcin suppresses cell death in cloned rat hepatoma H4-II-E cells induced by tumor necrosis factor-α or thapsigargin. J Cell Biochem.

[B14] Fujita T, Shirasawa T, Maruyama N (1996). Isolation and characterization of genomic and cDNA clones encoding mouse senescence marker protein-30 (SMP30). Biochim Biophys Acta.

[B15] Yamaguchi M, Tsurusaki Y, Misawa H, Inagaki S, Ma ZJ, Takahashi H (2002). Potential role of regucalcin as a specific biochemical marker of chronic liver injury with carbon tetrachloride administration in rats. Mol Cell Biochem.

[B16] Isogai M, Shimokawa N, Yamaguchi M (1994). Hepatic calcium-binding protein regucalcin is released into the serum of rats administered orally carbon tetrachloride. Mol Cell Biochem.

[B17] Ishigami T, Fujita T, Simbula G, Columbano A, Kikuchi K, Ishigami A, Shimosawa T, Arakawa Y, Maruyama N (2001). Regulatory effects of senescence marker protein 30 on the proliferation of hepatocytes. Pathol Int.

[B18] Isogai M, Oishi K, Yamaguchi M (1994). Serum release of hepatic calcium-binding protein regucalcin by liver injury with galactosamine administration in rats. Mol Cell Biochem.

[B19] Jung KJ, Ishigami A, Maruyama N, Takahashi R, Goto S, Yu BP, Chung HY (2004). Modulation of gene expression of SMP-30 by LPS and calorie restriction during aging process. Exp Gerontol.

[B20] Yamaguchi M, Daimon Y (2005). Overexpression of regucalcin suppresses cell proliferation in cloned rat hepatoma H4-II-E cells: involvement of intracellular signaling factors and cell cycle-related genes. J Cell Biochem.

